# The effect of *NOTCH3* pathogenic variant position on CADASIL disease severity: *NOTCH3* EGFr 1–6 pathogenic variant are associated with a more severe phenotype and lower survival compared with EGFr 7–34 pathogenic variant

**DOI:** 10.1038/s41436-018-0088-3

**Published:** 2018-07-22

**Authors:** Julie W. Rutten, Bastian J. Van Eijsden, Marco Duering, Eric Jouvent, Christian Opherk, Leonardo Pantoni, Antonio Federico, Martin Dichgans, Hugh S. Markus, Hugues Chabriat, Saskia A. J. Lesnik Oberstein

**Affiliations:** 1 CADASIL Research Group, Department of Clinical Genetics, Leiden University Medical Center, Leiden, The Netherlands; 2 Department of Human Genetics, Leiden University Medical Center, Leiden, The Netherlands; 3 Institute for Stroke and Dementia Research, University Hospital (LMU), Munich, Germany; 4 Department of Neurology, AP-HP, Lariboisière Hospital, Paris, France; 5 Department of Neurology, SLK-Kliniken Heilbronn, Heilbronn, Germany; 6 “L. Sacco” Department of Biomedical and Clinical Sciences, University of Milan, Milan, Italy; 7 Department of Medicine, Surgery and Neurosciences, Medical School, University of Siena, Siena, Italy; 8 Stroke Research Group, Department of Clinical Neurosciences, University of Cambridge, Cambridge, UK

**Keywords:** CADASIL, Genotype–phenotype correlation, NOTCH3, Small-vessel disease

## Abstract

**Purpose:**

CADASIL is a small-vessel disease caused by a cysteine-altering pathogenic variant in one of the 34 epidermal growth factor-like repeat (EGFr) domains of the NOTCH3 protein. We recently found that pathogenic variant in EGFr domains 7–34 have an unexpectedly high frequency in the general population (1:300). We hypothesized that EGFr 7–34 pathogenic variant more frequently cause a much milder phenotype, thereby explaining an important part of CADASIL disease variability.

**Methods:**

Age at first stroke, survival and white matter hyperintensity volume were compared between 664 CADASIL patients with either a *NOTCH3* EGFr 1–6 pathogenic variant or an EGFr 7–34 pathogenic variant. The frequencies of NOTCH3 EGFr 1–6 and EGFr 7–34 pathogenic variant were compared between individuals in the genome  Aggregation Database and CADASIL patients.

**Results:**

CADASIL patients with an EGFr 1–6 pathogenic variant have a 12-year earlier onset of stroke than those with an EGFr 7–34 pathogenic variant, lower survival, and higher white matter hyperintensity volumes. Among diagnosed CADASIL patients, 70% have an EGFr 1–6 pathogenic variant, whereas EGFr 7–34 pathogenic variant strongly predominate in the population.

**Conclusion:**

*NOTCH3* pathogenic variant position is the most important determinant of CADASIL disease severity, with EGFr 7–34 pathogenic variant predisposing to a later onset of stroke and longer survival.

## INTRODUCTION

CADASIL (cerebral autosomal dominant arteriopathy with subcortical infarcts and leukoencephalopathy) is the most prevalent hereditary cerebral small-vessel disease, caused by highly distinctive cysteine-altering missense pathogenic variant (PV) in the *NOTCH3* gene.^1,2^ More than 200 of such distinct *NOTCH3* PV have been described in CADASIL families worldwide.^3^ Recently, we have shown that these PV occur in 1:300 individuals in the general population worldwide, which is 100-fold higher than the minimal estimated CADASIL prevalence.^4^ This unexpectedly high frequency not only supports the hypothesis that CADASIL is underdiagnosed, but also strongly suggests that, in the general population, these PV are probably associated with a much milder small-vessel disease phenotype, or may sometimes even be non-penetrant.

Although *NOTCH3* PV in the population are identical to those found in CADASIL patients, we previously found that the PV in the general population are predominantly located in epidermal growth factor-like repeat (EGFr) domains 7–34 of the NOTCH3 protein, whereas PV in CADASIL patients are predominantly located in EGFr domains 1–6. This suggests that EGFr domain 1–6 PV are associated with a more severe or ‘classical’ CADASIL phenotype, whereas EGFr 7–34 PV are generally milder. Indeed, in a small CADASIL cohort we previously showed that EGFr domain 1-6 PV are associated with a higher brain lesion load on  magnetic resonance imaging (MRI), than EGFr domain 7–34 PV.^4^ As such, the *NOTCH3* PV position may be an important, hitherto unrecognized, factor underlying CADASIL disease variability—possibly much more so than the previously identified vascular risk factors and genetic modifiers, which account for only a small part of the disease variability.^5-8^ Classically, CADASIL patients have their first stroke at age 45–50 years^9^ and develop progressive cognitive impairment leading to vascular dementia. Life expectancy is reduced, and has been estimated at 64.6 years for males and 70.7 years for females.^10^ Other frequent symptoms are migraine with aura, mood disturbances and apathy.^11^ Typical brain MRI abnormalities are progressive symmetrical white matter hyperintensities (WMHs), lacunes, microbleeds and brain atrophy.^12–14^ Disease variability is increasingly recognized, especially at the milder end, with patients with a much later disease onset well into the eighth decade.^4,15,16^

In the current study, we aimed to validate and further delineate the effect of *NOTCH3*  PV position on CADASIL disease variability, by analysing the correlation between PV position and brain MRI lesion load, age at first stroke and survival in two independent large CADASIL samples.^4^ Furthermore, we compared the locations of *NOTCH3* PV in European CADASIL patients with those in the general European population, using the  genome  Aggregation Database (gnomAD).

## MATERIALS AND METHODS

### Analysis of disease severity and survival in 251 Dutch CADASIL patients

The files of 251 patients in the Dutch CADASIL registry who visited the Leiden University Medical Center between 1998 and 2017 were evaluated. In all patients, CADASIL had been confirmed by the detection of an EGFr domain cysteine-altering *NOTCH3* PV. Individuals had either had diagnostic DNA testing after a clinical diagnosis, or had requested predictive genetic testing due to a positive family history for CADASIL. Only individuals with a heterozygous PV were included in the study. A patient with a compound heterozygous PV and a patient with a homozygous PV were therefore excluded. Patients originated from 131 unrelated families, with 45 distinct PV in 16 discrete EGFr domains. Of these patients, 153 had a PV in one of EGFr domains 1–6 and 98 had a PV in one of EGFr domains 7–34. Medical records were examined by an investigator blinded to the location of the PV, who recorded the date of birth, sex and family structure, occurrence and age at  onset of first stroke, and presence or absence of migraine with aura, as well as cardiovascular risk factors including history of smoking, hypertension (systolic (>140 mmHg) or diastolic (>90 mmHg), or antihypertensive treatment), and previous diagnosis of diabetes mellitus. Stroke was defined as an episode of acute neurological deficits lasting longer than 24 h. If a brain MRI scan was available, stroke was verified. Migraine with aura was defined as an episode of typical visual aura, or visual aura followed or accompanied by spreading sensory disturbances (tingling or numbness) in a limb or verbal dysphasia, lasting between 10 and 30 min, followed (or not) by migraine headache. If events were difficult to classify, they were independently assessed by two experienced physicians and classified by consensus. Hypercholesterolaemia could not be assessed accurately from the patient records because, according to international guidelines,^17^ cholesterol-lowering agents are prescribed to all patients who have had a stroke as a preventative measure. This was therefore not included in the analysis. Survival data were obtained from the Dutch population registry. The study was approved by the Medical Ethics Committee of the Leiden University Medical Center (G17.073).

### Analysis of brain MRI WMH volume in 412 European CADASIL patients

The European CADASIL sample included 470 CADASIL patients, recruited from 5 European countries (France, Germany, United Kingdom, Italy and the Netherlands), in whom brain MRI WMH volumes had been previously quantified.^7^ Data on the age at first stroke and survival were not available for this sample. We excluded from the analysis the 51 patients from the Netherlands, as this was the discovery sample of the genotype–phenotype correlation described in a previous study.^4^ We also excluded six patients who did not have a missense PV,^18,19^ and one patient whose age was not known. A total of 412 patients were included in the final analysis. In this sample, there were 97 distinct PV in 21 discrete EGFr domains. Of the 412 patients, 290 had a PV in one of EGFr domains 1–6 and 122 had a PV in one of EGFr domains 7–34. For all patients, date of birth, sex, country of origin, brain MRI WMH volume, *APOE* carrier status, and cardiovascular risk factors including smoking status, number of pack years (multiplication of number of cigarette packs smoked per day with number of years of smoking), hypertension, hypercholesterolaemia and diabetes mellitus, were registered in a database. *APOE* carrier status was grouped based on the presence of the ϵ2 and ϵ4 allele.^8^ For 35 individuals, the *APOE* genotype was not known. These individuals were coded as a separate group. Details on brain MRI processing and quantification have been described previously.^7^ Briefly, brain MRI scans were performed at each site, at field strengths between 0.5 and 3 Tesla. WMH volumes were measured on fluid attenuated inversion recovery (FLAIR) sequences using a semi-automated method, where all hyperintense subcortical lesions on FLAIR imaging were labelled WMH. The intracranial cavity was assessed using an automated three-dimensional image-segmentation algorithm followed by manual correction. WMH volumes were divided by the intracranial cavity volume to normalize for head size. Given the left-skewed distribution of the WMH volume, the square root was taken to obtain a normal distribution. This is referred to as the normalized WMH volume (nWMHV).

### Analysis of *NOTCH3* PV in the general population using gnomAD

We previously found an unexpectedly high frequency of EGFr cysteine-altering *NOTCH3* PV in a  large exome database, the Exome Aggregation Consortium (ExAC).^20^ This database has now been expanded and renamed gnomAD (http://gnomad.broadinstitute.org/). GnomAD includes more than double the number of individuals in ExAC, with 123,136 exome sequences and 15,496 whole-genome sequences from unrelated individuals sequenced as part of various disease-specific and population genetic studies.^20^ We queried gnomAD for archetypal CADASIL-causing PV; i.e., missense PV leading to the gain or loss of a cysteine residue in one  of the 34 EGFr domains of the NOTCH3 protein (amino acid residues 40–1373) (http://www.uniprot.org). To compare the PV location of individuals in gnomAD with the PV location found in diagnosed CADASIL patients, we selected only those individuals of European descent (76,266 individuals), and compared these with 463 diagnosed European CADASIL patients.^7^ For PV annotation, the reference sequence NM_000435.2 was used, and sequence variants were described according to the Human Genome Variation Society nomenclature recommendations.

### Statistical analysis

Differences in baseline characteristics (age, sex and cardiovascular risk factors) were analysed using independent-sample *t*-tests for continuous variables and Chi-squared tests for categorical variables. Differences between the prevalence of stroke and migraine with aura in patients with a PV in EGFr domains 1–6 versus patients with a PV in EGFr domains 7–34 were analysed using Chi-squared tests. Differences in the age of onset of first stroke and survival were analysed through time-to-event analyses and log-rank tests, as this also incorporates data from individuals who had not yet had an event (stroke or death) at the time of the last follow-up (right censoring). Correction for age, sex and cardiovascular risk factors was performed using Cox-regression analysis. To account for a possible effect caused by multiple members of a single family, a sensitivity analysis including only one randomly selected member per family was performed. The association between the location of the *NOTCH3* PV and nWMHV was performed using stepwise linear regression with forward selection. nWMHV was entered as a dependent variable, and country, *APOE* genotype, age, sex, hypertension, hypercholesterolaemia, diabetes and smoking were entered as independent variables. Mutation location was grouped into EGFr 1–6 and EGFr 7–34, and EGFr group was included as an additional independent variable. For all analyses, the *⍺* level (i.e. the significance level) was set at 5%.

## RESULTS

### Mutations in *NOTCH3* EGFr domains 1–6 are associated with an earlier onset of stroke than PV in EGFr domains 7–34

**Fig. 1:**
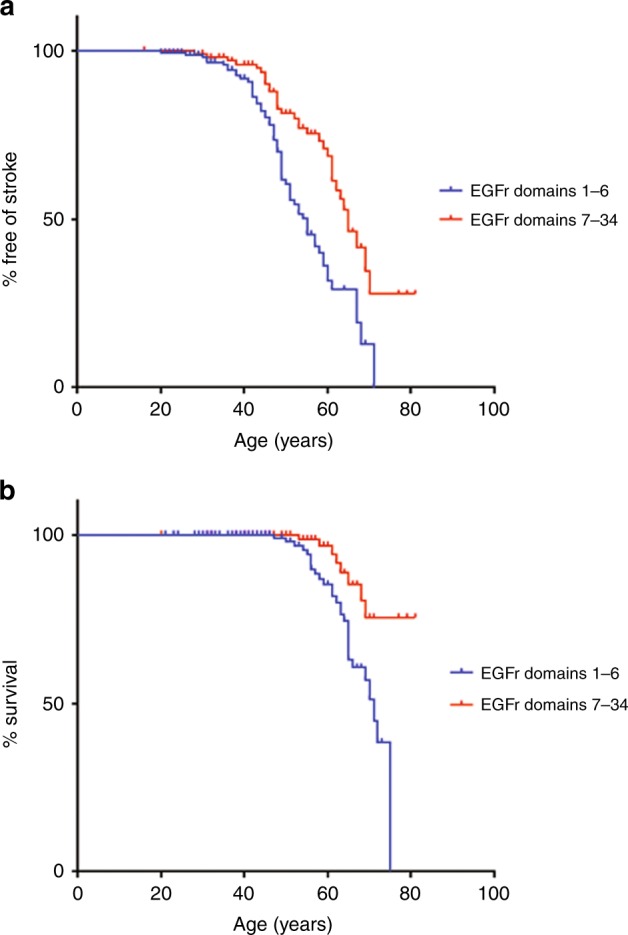
Mutations in *NOTCH3*  EGFr domains 1–6 are associated with an earlier age of onset of stroke and lower life expectancy than PV in EGFr domains 7–34. **a**, **b**, Kaplan–Meier plots showing the differences in (**a**) age of onset of first stroke and (**b**) survival stratified according to mutation position for patients with epidermal growth factor-like repeat (EGFr) domain 1–6 PV versus those with EGFr domain 7–34 PV

First, we compared the age at onset of stroke in the 153 Dutch CADASIL patients with an EGFr 1–6 PV with the 98 patients with an EGFr 7–34 PV. At the time of the DNA test, patients with an EGFr domain 1–6 PV were on average 8.2 years younger than patients with an EGFr 7–34 PV (44.3 years versus 52.5 years, *P* < 0.001, independent-samples *t*-test) (Table 1), suggesting that patients with a PV in EGFr domains 1–6 are diagnosed at a younger age. In the group of patients with an EGFr 1–6 PV, 41.2% had experienced at least one stroke, compared to 30.6% of patients with an EGFr 7–34 PV (*P* = 0.091, Chi-squared test). In time-to event-analysis, median latencies until first stroke were age 55 for patients with an EGFr 1–6 PV and age 67 for those with an EGFr 7–34 PV (*P* < 0.001, log-rank test) (Fig. 1a). After correction for sex and cardiovascular risk factors, the location of the PV was the highest predictive covariate for age at first stroke, with a hazard ratio (HR) for EGFr 1–6 PV versus EGFr 7–34 PV of 2.63 (95% confidence interval (CI) = 1.61–4.31, *P* < 0.001, Cox-regression analysis). There was no significant effect of hypertension or smoking status (HR = 1.45, 95% CI = 0.90–2.30, *P* = 0.121; and HR = 1.74, 95% CI = 0.63–1.56, *P* = 0.97, respectively). The effect of *NOTCH3* PV position on age at first stroke was not influenced by family of origin (i.e., when including only one individual per family, similar results were found). Prevalence and age at onset of migraine with aura did not differ between patients with an EGFr 1–6 PV and patients with an EGFr 7–34 PV (35.6 versus 32.6%, *P* = 0.638, Chi-squared test; and 30.6 versus 26.6 years, *P* = 0.24, independent-samples *t-*test, respectively).

**Table 1:** Characteristics of patients in the Dutch CADASIL sample

	EGFr 1–6	EGFr 7–34	*P* *value*
*n*	153	98	
Mean age at DNA test (years (95% CI))	44.3 (42.3–46.3)	52.5 (50.2–54.8)	<0.001
Male/female (% male)	69/84 (45.1%)	42/56 (42.9%)	0.727
Smoking, yes/no (%)	69/75 (47.9%)	50/35 (58.8%)	0.110
	9 unknown	13 unknown	
Hypertension, yes/no (%)	30/114 (20.8%)	33/54 (37.9%)	0.005
	12 unknown	11 unknown	
Diabetes, yes/no (%)	11/134 (7.6%)	6/79 (7.1%)	0.883
	8 unknown	13 unknown	

*EGFr* Epidermal growth factor-like repeat

### Correlation between *NOTCH3* PV position and survival

Next, we compared survival rates between patients with a *NOTCH3* EGFr 1–6 PV and patients with an EGFr 7–34 PV. At the end of 2017, 28 out of 153 Dutch patients with an EGFr 1–6 PV were deceased (18.3%), compared with 8 out of 98 patients with an EGFr 7–34 PV (8.2%) (*P* = 0.025, Chi-squared test). The mean survival time was 68.5 years for patients with an EGFr 1–6 PV, compared with 76.9 years for patients with an EGFr 7–34 PV (*P* = 0.004, log-rank test) (Fig. 1b). After correction for sex and cardiovascular risk factors, the location of the PV was the highest predictive covariate for survival (HR = 3.11, 95% CI = 1.16–8.34, *P* = 0.024, Cox-regression analysis).

###  Mutations in *NOTCH3*  EGFr domains 1–6 are associated with a higher WMH volume than PV in  EGFr domains 7–34

**Fig. 2:**
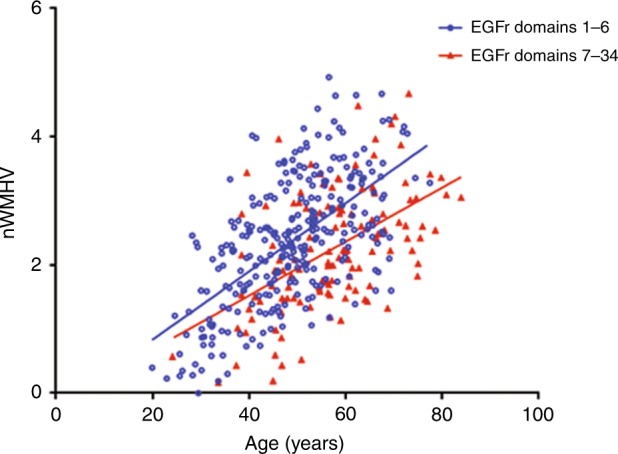
Mutations in *NOTCH3*  EGFr domains  1–6 are associated with a higher nWMHV than mutations in  EGFr domains  7–34 . Scatterplot showing the correlation between normalized white matter hyperintensity volume (nWMHV) and age, stratified according to epidermal growth factor-like repeat (EGFr) group. Diagnosed CADASIL patients with a mutation in one of EGFr domains 1–6 have a higher nWMHV than patients with a mutation in one of EGFr domains 7–34

We previously found a correlation between *NOTCH3* PV position and brain MRI lesion load in a small cohort of Dutch CADASIL patients.^4^ To validate these findings, here, we analysed the relationship between the position of the *NOTCH3* PV and nWMHV on brain MRI in an independent sample of 412 European CADASIL patients.^7^ At the time of their MRI scan, patients with a PV in EGFr domains 1–6 were younger than patients with a PV in EGFr domains 7–34 (48.8 versus 57.3 years, *P* < 0.001) (Table 2). Using stepwise regression, we found a significant association between PV location and nWMHV, where EGFr 1–6 PV are associated with a higher nWMHV than EGFr 7–34 PV (*β* = −0.144, *t* = −3.180, *P* = 0.002) (Fig. 2). There was a small difference in the age-dependent increase in nWMHV for individuals with an EGFr 1–6 PV versus individuals with an EGFr 7–34 PV, but this difference did not reach statistical significance (*P* = 0.132). Finally, we looked specifically at a possible effect of PV in the ligand-binding domain (EGFr 10–11). Interestingly, despite the overall effect of a lower nWMHV in the higher-numbered EGFr domains, we found that the average nWMHV in the 14 individuals with an EGFr 10–11 PV was higher than the average nWMHV in the rest of the sample (*β* = −0.122, *t* = −3.220, *P* = 0.001) (Figure S1).

**Table 2:** Characteristics of patients in the European CADASIL sample

	EGFr 1–6	EGFr 7–34	*P* *value*
*n*	290	122	
Mean age (years (95% CI))	48.8 (47.5–50.1)	57.3 (55.3–59.4)	<0.001
Male/female (% male)	130/160 (44.8)	55/67 (45.1)	0.962
Smoking, yes/no (%)	84/206 (29.0)	18/104 (14.8)	0.002
Pack years	5.7 (4.2–7.2)	13.0 (−0.4–26.3)	0.11
Hypercholesterolaemia, yes/no (%)	129/161 (44.5)	63/59 (51.6)	0.184
Hypertension, yes/no (%)	45/245 (15.5)	38/84 (31.1)	<0.001
Diabetes, yes/no (%)	6/284 (2.1)	3/119 (2.5)	0.728

*EGFr* Epidermal growth factor-like repeat

### EGFr domain 7–34 PV are much more prevalent in the general population than EGFr domain  1–6 PV

**Fig. 3:**
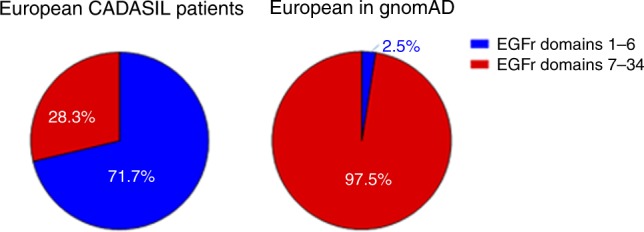
Location of *NOTCH3* mutations between diagnosed CADASIL patients and individuals in the gnomAD database. Pie charts displaying the differences in the number of mutations located in EGFr domains 1–6 versus 7–34, between European CADASIL patients and European individuals with a *NOTCH3* mutation in gnomAD

Finally, we compared the location of EGFr cysteine-altering *NOTCH3* missense PV between a large population sample (European individuals in gnomAD, *n* = 76,266) and the 463 patients included in the European CADASIL sample. In gnomAD, there were 450 individuals with a cysteine-altering *NOTCH3* PV (Figure S2). This corresponds to a frequency of 3.2 per 1000 individuals, which is in line with our previous findings in the ExAC database.^4^ Of the 450 individuals with a *NOTCH3* PV in gnomAD, 120 individuals were of European descent. Only a small minority of these had a PV in EGFr domains 1–6 (2.5%). In contrast, most of the patients in the European CADASIL sample had a PV in EGFr domains 1–6 (71.1%) (Fig. 3). This shows that, although cysteine-altering *NOTCH3* PV are distributed along the 34 EGFr domains in both CADASIL patients and the general population, in diagnosed CADASIL patients the PV are predominantly located in one of EGFr domains 1–6, whereas in the general population the PV are predominantly located in one of EGFr domains 7–34. This strongly suggests that EGFr 7–34 PV predispose to a much milder phenotype, which we also show to be the case even within diagnosed CADASIL patients.

## DISCUSSION

This study shows that the PV position along the 34 EGFr domains of the NOTCH3 protein plays an important role in the *NOTCH3* disease spectrum, with EGFr domain 1–6 PV predisposing to ‘classical’ CADASIL, while EGFr domain 7–34 PV predispose to a milder phenotype. We found that CADASIL patients with an EGFr domain 1–6 PV had a 12-year-earlier age at onset of stroke, lower survival time and greater brain MRI lesion load than patients with an EGFr domain 7–34 PV. We also found that the EGFr PV position is the strongest predictor of CADASIL disease severity after age. This PV position effect would probably be even stronger if the phenotype of individuals in the population were included in a genotype–phenotype correlation study, as the vast majority of individuals with a cysteine-altering *NOTCH3* PV in the general population have an EGFr domain 7–34 PV.

We have extrapolated that of all individuals with an EGFr cysteine-altering *NOTCH3* PV, only approximately 1% are diagnosed with CADASIL (i.e., most individuals with these PV reside in the general population, rather than in the diagnosed CADASIL population). This is a striking finding as, to date, these distinctive EGFr cysteine-altering *NOTCH3* PV have been considered to be specific for CADASIL and confirmative of a diagnosis. We speculate that *NOTCH3 *EGFr 7–34 PV in a population sample could well be associated with attenuated CADASIL or a late-onset small-vessel disease phenotype, which may be largely indistinguishable from what is currently considered to be a ‘normal’ cerebrovascular ageing process (i.e., an elderly onset of mild cognitive deficits, ischaemic events and some degree of WMHs on MRI). This hypothesis is supported by the increasing number of CADASIL patients who are diagnosed above 65 years of age, showing that even within known CADASIL families, cysteine-altering *NOTCH3* PV can be associated with a relatively mild, later-onset phenotype.^4,15,16^ From the population perspective, it is therefore possible that cysteine-altering *NOTCH3* PV in higher EGFr domains are a hitherto overlooked risk factor for cerebral small-vessel disease in the (elderly) population. Whether PV in higher-numbered EGFr domains can be non-penetrant remains to be determined.

Cardiovascular and other genetic risk factors have previously been described to contribute to CADASIL disease severity.^5^ Possibly, EGFr 7–34 PV require a higher additional (vascular) risk-factor profile before causing CADASIL or a small-vessel disease phenotype, whereas an EGFr 1–6 PV alone almost always predisposes to a ‘classical’ CADASIL phenotype. Indeed, in both the European and Dutch CADASIL patients, the prevalence of hypertension was higher in patients with an EGFr 7–34 PV, even after correcting for age. *NOTCH3* PV position and traditional vascular risk factors are probably only the first in a list of players determining *NOTCH3* disease severity, considering the fact that individuals with the same PV, or even within the same pedigree, can show variability in disease onset and progression. In our study, we found that disease severity was more strongly determined by *NOTCH3* PV position than by hypertension or smoking. Taken together, it is important to include PV position in future *NOTCH3* disease prediction models, which need to be developed to help predict disease severity in newly diagnosed CADASIL patients, and especially in individuals in whom a cysteine-altering *NOTCH3* PV is revealed as a chance finding through exome or genome sequencing.

We can only speculate as to the mechanisms underlying the *NOTCH3* PV position effect, which may lie anywhere from protein expression and processing to protein interactions and differences in mutant NOTCH3 aggregation properties. Interestingly, despite an overall effect of higher-numbered EGFr domains being associated with a milder phenotype, we found that PV in EGFr domains 10 and 11, which comprise the ligand-binding domain and lead to reduced NOTCH3 signalling,^21–24^ are associated with a higher WMH lesion load when compared with all other EGFr domains. We could not assess the association of EGFr 10–11 PV with age at first stroke or survival because the number of patients with a PV in the ligand-binding domain in the Dutch CADASIL sample was too low for reliable analysis. Previous studies have reported contradictory findings regarding patients with a ligand-binding domain PV; both a milder and more severe phenotype have been described.^22,25^ Larger studies including more phenotypic information are needed to determine whether there is an additional effect of specific EGFr domains, including the ligand-binding domain.

In conclusion, we show that CADASIL patients with an EGFr domain 1–6 PV have a more severe CADASIL phenotype than patients with an EGFr domain 7–34 PV, where EGFr 7–34 PV are by far the most common in the general population. These findings strongly suggest that the presence of an EGFr 1–6 PV alone predisposes to the classical, more severe CADASIL phenotype, whereas the EGFr 7–34 disease spectrum is much broader, ranging from an attenuated CADASIL phenotype to (possibly) non-penetrance. This completely transforms the CADASIL–NOTCH3 disease paradigm, as patients who are currently diagnosed with CADASIL are merely the tip of the iceberg of individuals with a *NOTCH3* PV, and probably represent only the severe end of the *NOTCH3* disease spectrum. Identifying these patients at risk for early-onset, severe disease will facilitate biomarker and clinical endpoint development, as well as appropriate patient selection and inclusion in future clinical trials.

The role of *NOTCH3* PV in the general population in relation to cerebral small-vessel disease will probably be clarified in the near future as large study cohorts become available, including not only exome- and genome-sequencing data, but also MRI and other clinical phenotyping. Discovering which factors protect the majority of individuals harbouring such PV from developing a classical CADASIL phenotype will probably lead to new insights for disease prevention, and possibly also to the identification of targets for therapeutic intervention.

## Electronic supplementary material

Supplementary Information

## SUPPLEMENTARY MATERIAL

Supplementary material is linked to the online version of this paper at 10.1038/s41436-018-0088-3
