# Correction: The effect of NOTCH3 pathogenic variant position on CADASIL disease severity: NOTCH3 EGFr 1–6 pathogenic variant are associated with a more severe phenotype and lower survival compared with EGFr 7–34 pathogenic variant

**DOI:** 10.1038/s41436-018-0306-z

**Published:** 2018-09-20

**Authors:** Julie W. Rutten, Bastian J. Van Eijsden, Marco Duering, Eric Jouvent, Christian Opherk, Leonardo Pantoni, Antonio Federico, Martin Dichgans, Hugh S. Markus, Hugues Chabriat, Saskia A. J. Lesnik Oberstein

**Affiliations:** 1 CADASIL Research Group, Department of Clinical Genetics, Leiden University Medical Center, Leiden, The Netherlands; 2 Department of Human Genetics, Leiden University Medical Center, Leiden, The Netherlands; 3 Institute for Stroke and Dementia Research, University Hospital (LMU), Munich, Germany; 4 Department of Neurology, AP-HP, Lariboisière Hospital, Paris, France; 5 Department of Neurology, SLK-Kliniken Heilbronn, Heilbronn, Germany; 6 “L. Sacco” Department of Biomedical and Clinical Sciences, University of Milan, Milan, Italy; 7 Department of Medicine, Surgery and Neurosciences, Medical School, University of Siena, Siena, Italy; 8 Stroke Research Group, Department of Clinical Neurosciences, University of Cambridge, Cambridge, UK

Correction to: *Genetics in Medicine* ; 10.1038/s41436-018-0088-3; published online 22 July 2018

This Article was originally published under Nature Research’s License to Publish, but has now been made available under a [CC BY 4.0] license. The PDF and HTML versions of the Article have been modified accordingly.

