# Supplementary material for: The effect of NOTCH3 pathogenic variant position on CADASIL disease severity: NOTCH3 EGFr 1–6 pathogenic variant are associated with a more severe phenotype and lower survival compared with EGFr 7–34 pathogenic variant
Source: Genet Med. 2018 Jul 22;21(3):676–82. doi: 10.1038/s41436-018-0088-3 (PMC6752295; doi:10.1038/s41436-018-0088-3)
Supplement: Supplementary file 1 — Supplementary Information [file 41436_2018_88_MOESM1_ESM.docx]

***Figure S1. Patients with a mutation in the NOTCH3 ligand binding domain have a relatively high white matter hyperintensity lesion load.*** *Scatterplot* *showing the correlation between normalized white matter hyperintensity volume (nWMHV) and age, for patients with a mutation in the NOTCH3 ligand binding domain compared to all other mutations.*

***
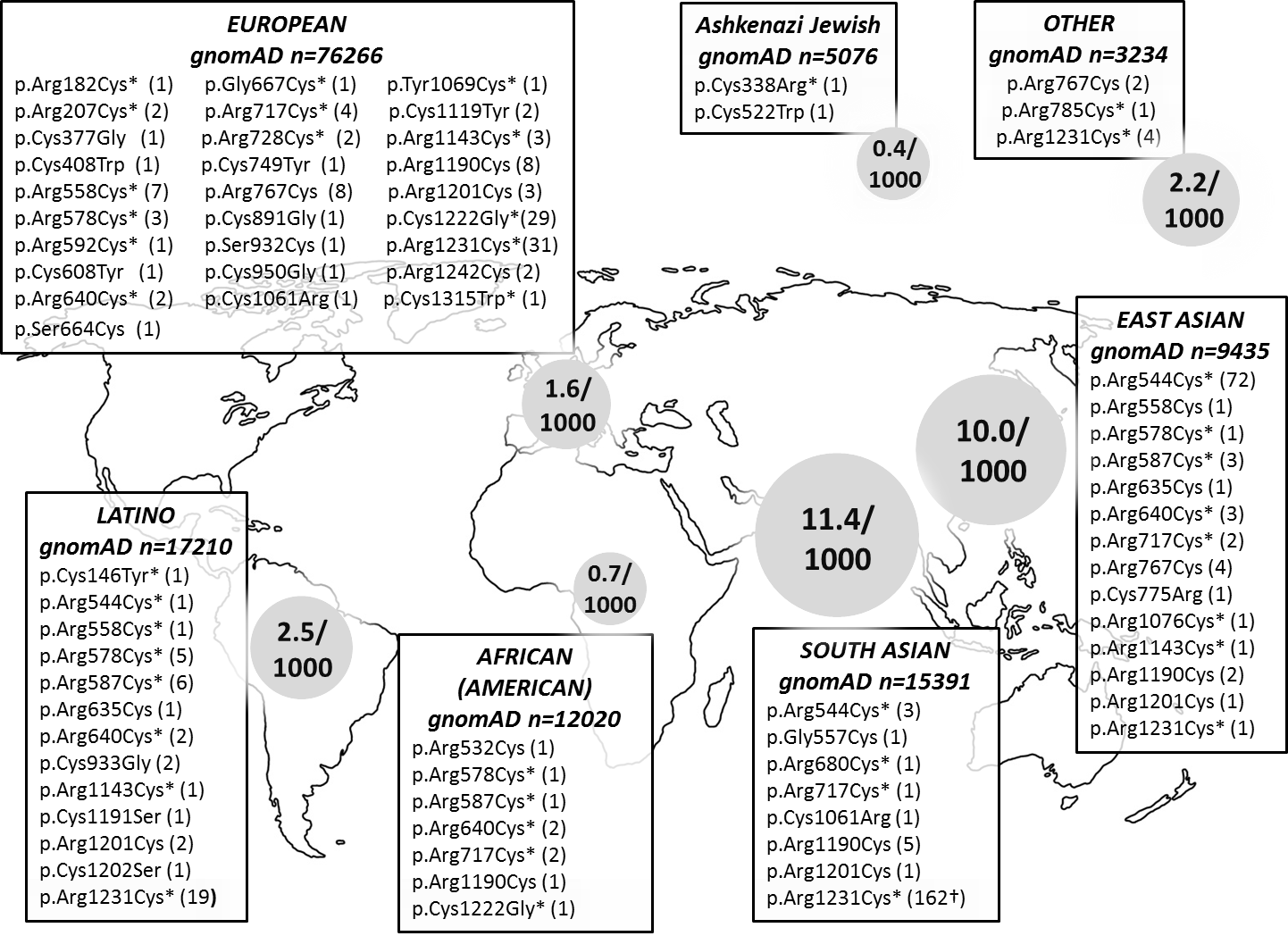
***

***Figure S2. NOTCH3 mutations in the gnomAD database, according to ethnicity.*** *Frequencies range from 0.4/1000 individuals in the Ashkenazi Jewish population to 1.6/1000 in the European population and 11.4/1000 in the South Asian population, with an overall frequency of 3.2/1000. NOTCH3 mutation frequencies in gnomAD are largely similar to those previously found in the ExAC database. Figure adapted and updated from Rutten et al. ACTN 2016.^1^*

1. Rutten JW, Dauwerse HG, Gravesteijn G, et al. Archetypal NOTCH3 mutations frequent in public exome: implications for CADASIL. *Annals of clinical and translational neurology.* 2016;3(11):844-853.
